# Phylogenetic Tracking of LA-MRSA ST398 Intra-Farm Transmission among Animals, Humans and the Environment on German Dairy Farms

**DOI:** 10.3390/microorganisms9061119

**Published:** 2021-05-21

**Authors:** Tobias Lienen, Arne Schnitt, Christiane Cuny, Sven Maurischat, Bernd-Alois Tenhagen

**Affiliations:** 1 Department Biological Safety, German Federal Institute for Risk Assessment (BfR), 10589 Berlin, Germany; Arneschnitt@posteo.de (A.S.); sven.maurischat@bfr.bund.de (S.M.); 2 Department Infectious Diseases, Robert-Koch Institute (RKI), 38855 Wernigerode, Germany; Cunych@rki.de

**Keywords:** LA-MRSA, dairy farms, intra-farm transmission, phylogenomics

## Abstract

Methicillin-resistant *Staphylococcus* *aureus* (MRSA) are a major threat to human and animal health, causing difficult-to-treat infections. The aim of our study was to evaluate the intra-farm transmission of livestock-associated (LA) MRSA sequence type (ST) 398 isolates on German dairy farms. A total of 115 LA-MRSA ST398 isolates originating from animals, humans and the environment of six dairy farms were analyzed by whole-genome sequencing and core genome multilocus sequence typing. Phylogenetic clusters of high allelic similarity were detected on all dairy farms, suggesting a MRSA transmission across the different niches. On one farm, closely related isolates from quarter milk samples (QMS), suckers of calf feeders and nasal cavities of calves indicate that MRSA may be transferred by feeding contaminated milk to calves. Detection of related MRSA isolates in QMS and teat cups (4/6 farms) or QMS and human samples (3/4 farms) pointed out a transmission of MRSA between cows during the milking process and a potential zoonotic risk. In conclusion, LA-MRSA ST398 isolates may spread between animals, humans and the environment on dairy farms. Milking time hygiene and other internal biosecurity measures on farms and pre-treatment of milk before feeding it to calves may reduce the risk of MRSA transmission.

## 1. Introduction

Methicillin-resistant *Staphylococcus aureus* (MRSA) have been frequently found on dairy farms [1,2,3]. Infections such as bovine mastitis caused by MRSA are difficult to treat due to their resistance to virtually all beta-lactam antibiotics, a class of antibiotics that is commonly used for mastitis treatment [4]. In particular, livestock-associated (LA-) MRSA belonging to the sequence type (ST) 398 have been detected in livestock farms. Moreover, LA-MRSA ST398 have been shown to be transmitted to humans via animal contact [5,6,7]. However, the risk for severe infections in humans caused by LA-MRSA ST398 is low, since the repertoire of virulence associated genes that are connected to a harm of human health is generally low [8,9]. Nonetheless, with respect to dairy herd health, LA-MRSA ST398 may cause infections such as bovine mastitis, which may negatively impact animal welfare and dairy farm profit [10]. Accordingly, several studies demonstrated the linkage of LA-MRSA detection in quarter milk samples (QMS), and increased somatic cell counts, which is a common indicator for an inflammatory process in the cows’ udder [11,12]. Therefore, it is crucial to prevent the spread of LA-MRSA on dairy farms. Since LA-MRSA ST398 have not only been found in QMS and bulk tank milk (BTM), but also in calves, heifers, the farm environment as well as in farm workers [11,13,14], monitoring of MRSA presence in different sample types across dairy farms is necessary. Thus far, in most studies, the distinction of MRSA isolates, which are distributed across livestock farms, is based on the association of the *spa* and/or SCC*mec* type [15,16,17,18]. However, whole-genome sequencing (WGS) and subsequent phylogenetic analyses based on a comparison of a high number of allele sequences seem to be a more appropriate method to investigate the transmission of clonal isolates on dairy farms [9,19,20].

Therefore, the aim of the present study was to evaluate the intra-farm transmission of LA-MRSA ST398 isolates between animals, humans and the environment on six German dairy farms in depth and to give recommendations for transmission prevention. For this, WGS of LA-MRSA ST398 isolates originating from different samples across the dairy farms was conducted and phylogenetic analyses using core genome multilocus sequence typing (cgMLST) was performed to investigate the clonality of the isolates.

## 2. Materials and Methods

### 2.1. Sampling and MRSA Isolation

In our study, a total of 115 MRSA isolates from six German dairy farms, coded as A to F in the following, were obtained during a sampling campaign from September 2018 to December 2019. Once on each farm, QMS from 30 cows as well as swabs from 20 calves, ten heifers, pigs, farm personnel and from environmental niches such as milking equipment, automatic calf feeders and dust from barns were collected, if available. Moreover, one swab of an udder injury and a fluid sample from udder cleaning water was collected on one farm, respectively. Isolation of MRSA was based on a double selective enrichment method using cefoxitin. Sampling and MRSA isolation has been described in detail previously [11]. The size of the lactating cow herds on the farms and the number of MRSA isolates per farm are provided in Table 1.

### 2.2. DNA Extraction and WGS

MRSA isolates were inoculated in 5 mL brain–heart–infusion broth and incubated at 37 °C for 24 h. DNA of 1 mL culture was extracted using the Qiagen DNeasy Blood and Tissue Kit (Qiagen, Hilden, Germany) according to the manufacturer’s protocol modified by adding 10 µL lysostaphin (Sigma-Aldrich, St. Louis, MO, USA) to the lysis buffer. The DNA library was prepared using an Illumina Nextera DNA Prep kit (Illumina Inc., San Diego, CA, USA) and the 150 bp paired-end sequencing run was performed on an Illumina NextSeq 500 instrument (Illumina Inc., San Diego, CA, USA).

### 2.3. Bioinformatics and Phylogenetic Analyses

Raw Illumina reads were trimmed and de novo assembled with the in-house developed Aquamis pipeline (https://gitlab.com/bfr_bioinformatics/AQUAMIS/, accessed on 1 February 2021), which implements fastp [21] for trimming and shovill (based on Spades) (https://github.com/tseemann/shovill, accessed on 1 February 2021) for assembly. Furthermore, it performs mash v 2.1 for reference search [22] as well as quast v 5.0.2 for assembly quality control [23]. The minimal coverage depth was >30. Contamination control was done by single-copy and duplicated orthologs analyses. The fraction majority species was >0.97. The total genome length was >2.7 Mbp. The STs, *spa* types and phylogenetic relationship of all sequenced MRSA isolates were analyzed using cgMLST in Ridom SeqSphere+ version 7.0.4 (Ridom GmbH, Muenster, Germany) comparing 1861 alleles. Cluster generation was done according to default settings and defined as differences of ≤24 alleles according to *S*. *aureus* cgMLST scheme comparing 1861 alleles (https://www.cgmlst.org/ncs/schema/141106/, accessed on 10 March 2021) [24].

## 3. Results

### 3.1. Genotypic Characterization of LA-MRSA Isolates

**Figure 1:**
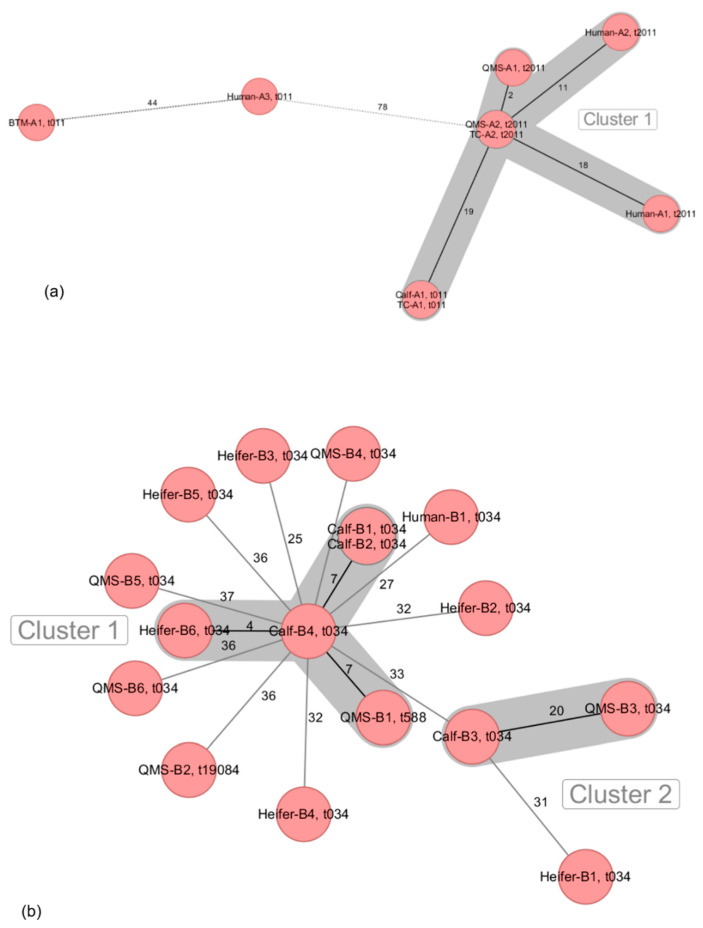
Minimum spanning tree visualization of cgMLST of LA-MRSA ST398 isolates from German dairy farms A (**a**) and B (**b**). Numbers give allelic differences between isolates. *spa* types are associated to every isolate. QMS = quarter milk sample; BTM = bulk tank milk; TC = teat cup.

**Figure 2:**
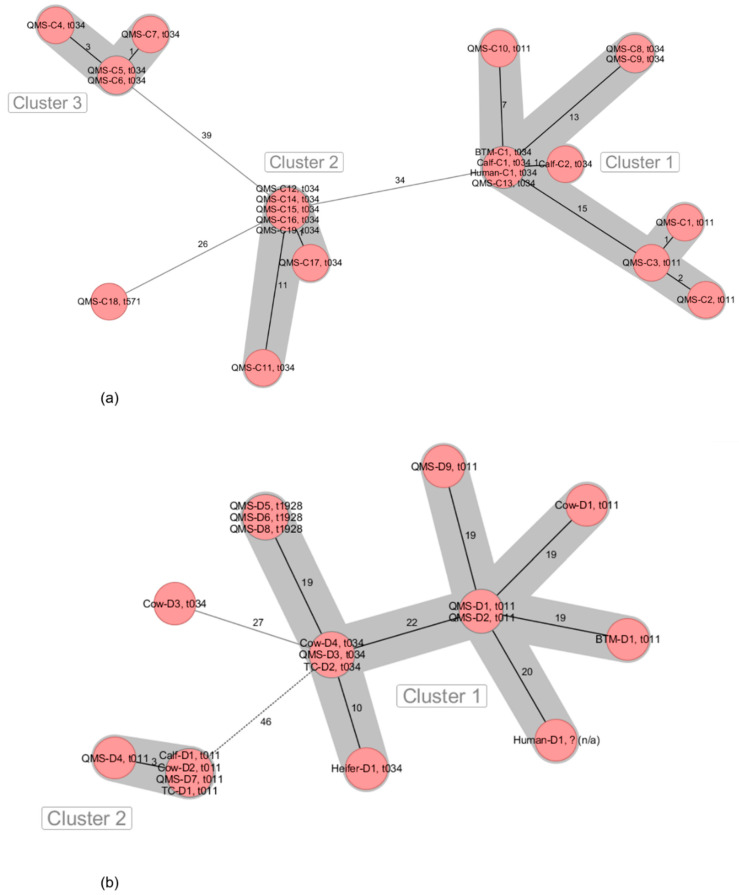
Minimum spanning tree visualization of cgMLST of LA-MRSA ST398 isolates from German dairy farms C (**a**) and D (**b**). Numbers give allelic differences between isolates. *spa* types are associated to every isolate. QMS = quarter milk sample; BTM = bulk tank milk; TC = teat cup.

**Figure 3:**
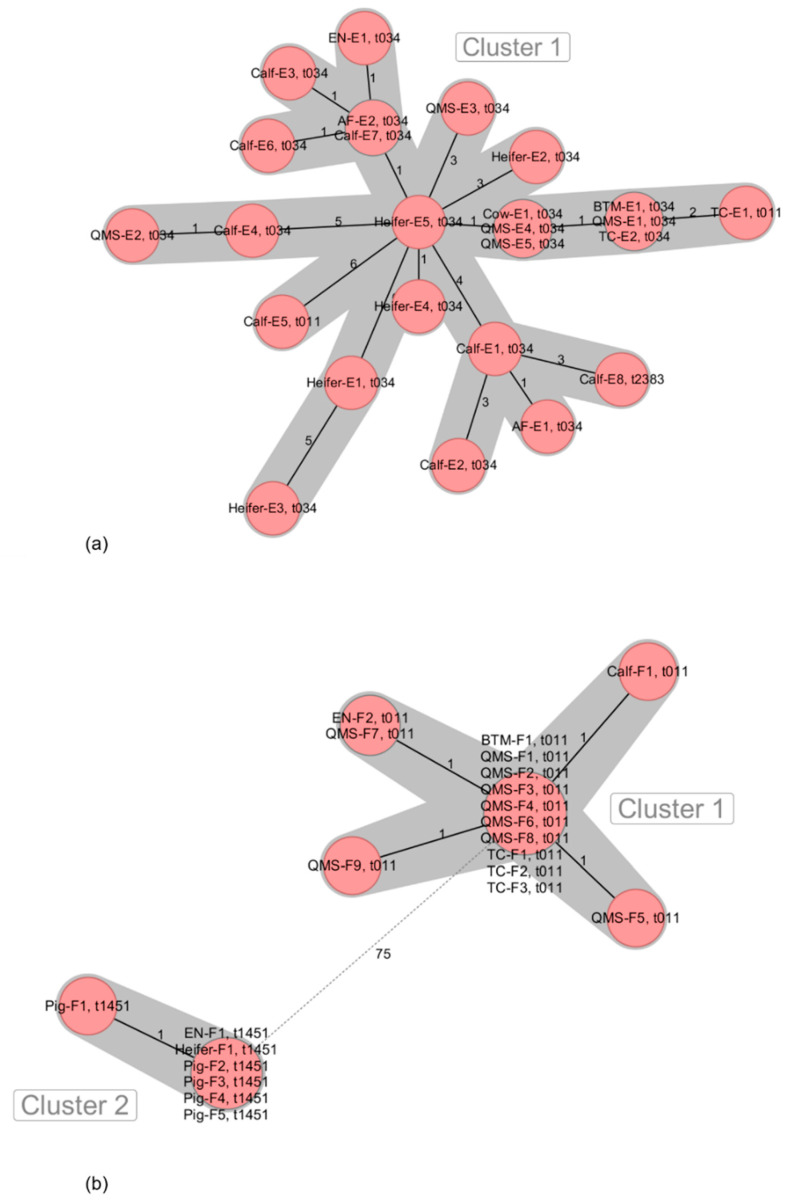
Minimum spanning tree visualization of cgMLST of LA-MRSA ST398 isolates from German dairy farms E (**a**) and F (**b**). Numbers give allelic differences between isolates. *spa* types are associated to every isolate. QMS = quarter milk sample; BTM = bulk tank milk; TC = teat cup, EN-E1 = dairy barn dust; EN-F1 = pig barn dust; EN-F2 = udder cleaning water; AF = automatic calf feeder.

MRSA isolates originated from QMS (6/6 farms), BTM (5/6 farms), calves (nasal swabs) (6/6 farms), heifers (nasal swabs and udder cleft swabs) (4/6 farms), pigs (nasal swabs) (1/6 farms) as well as environmental samples from the milking equipment (4/6 farms), automatic calf feeders (1/6 farm), dust from dairy (1/6 farms) and pig barns (1/6 farms) and nasal swab samples from farm personnel (4/6 farms) (Table 1). All MRSA from the six dairy farms were characterized as being LA-MRSA belonging to ST398. Several *spa* types were associated to the LA-MRSA on dairy farms A (t011, t2011) (Figure 1a), B (t034, t588, t19084) (Figure 1b), C (t011, t034, t571) (Figure 2a), D (t011, t034, t1928) (Figure 2b), E (t011, t034, t2383) (Figure 3a) and F (t011, t1451) (Figure 3b).

### 3.2. Intra-Farm Phylogenetic Relationship of LA-MRSA ST398 Isolates

The detected LA-MRSA ST398 isolates on the dairy farms varied in number (9 to 25) and source (Table 1). On farm A, nine LA-MRSA ST398 isolates were detected originating from BTM, QMS, teat cups (TC) of the milking equipment and nasal swabs of one calf and three farm workers. A cluster of close phylogenetic relationship was shown for QMS, TC, calf and two human samples, whereas LA-MRSA ST398 isolates from BTM and a third human sample showed clear genomic divergence to each other and all other LA-MRSA ST398 isolates (Figure 1a).

Partial genomic distinctions with allelic differences of >24 were also detected between most of the 17 LA-MRSA ST398 isolates from farm B. Two clusters of close phylogenetic relationship were observed within LA-MRSA ST398 originating from one QMS, three swabs from calves and a swab from one heifer as well as from one QMS and a swab of one calf, respectively (Figure 1b).

The LA-MRSA ST398 from farm C clustered into three separate groups. Cluster 1 was characterized by closely related LA-MRSA ST398 isolates from BTM, QMS, nasal swabs of two calves and a human nose sample, whereas clusters 2 and 3 showed closely related LA-MRSA ST398 isolates from seven and four different QMS, respectively (Figure 2a).

On farm D, 19 LA-MRSA ST398 isolates were grouped into two clusters with close relationship. LA-MRSA ST398 isolates from cluster 1 originated from BTM, QMS, TC and nasal swabs of one heifer, two cows and farm personnel (Figure 2b). Cluster 2 consisted of LA-MRSA ST398 isolates from QMS, TC and nasal swabs from one calf and one cow.

All 25 LA-MRSA ST398 isolates from farm E were closely related, resulting in only one phylogenetic cluster (Figure 3a). The isolates from this farm originated from BTM, QMS, TC, swabs from calves and heifers as well as from swabs from two automatic calf feeders (AF), dust from the dairy barn (EN) and a swab from an udder injury (Cow-E1).

The 22 LA-MRSA ST398 isolates from farm F grouped as two distantly related phylogenetic clusters (Figure 3b). Cluster 1 was characterized by LA-MRSA ST398 isolates from BTM, QMS, TC, a nasal swab from a calf and water for udder cleaning (EN-F2). In contrast, the LA-MRSA ST398 isolates associated to cluster 2 originated from nasal swabs from pigs, a dust sample from the pig barn (EN-F1) and a nasal swab from a heifer, which was kept in the pig barn.

## 4. Discussion

Mastitis in dairy cows is an important animal health issue and farmers suffer from economic losses caused by reduced milk yield and treatment costs [12]. LA-MRSA ST398 are often found to be involved in mastitis, thus, prevention and control measures are of high priority [4,10]. Since LA-MRSA ST398 may spread across different niches on dairy farms, it is crucial to track the transmission of clonal strains to recommend starting points for a prevention of their dissemination.

In our study, the transmission of LA-MRSA ST398 on six German dairy farms was studied in detail by WGS and phylogenetic analyses. Farm E gives a striking example for clonal LA-MRSA ST398 transmission on dairy farms. In total, 25 isolates from different sample types were detected on this farm and all of them showed a close relationship to each other with a maximal genotypic difference of only five alleles. This proves the spread of only one LA-MRSA ST398 strain across the whole dairy farm E. In contrast, several different LA-MRSA ST398 strains with various *spa* types were found on the other dairy farms. This is in line with previous reports from other groups [2,25] and may be based on independent introduction and further evolution of different LA-MRSA CC398 strains.

The close relationship of LA-MRSA ST398 in QMS and TC on farms A, D, E and F indicates a possible spread of these bacteria by contaminated TCs. Frequently, TCs are cleaned and disinfected after every milking process. However, in our study, LA-MRSA ST398 isolates were obtained from TCs of dairy farms that performed milking cluster disinfection on a regular basis. Obviously, contaminated TCs may be a source for transmission of LA-MRSA ST398 between cows and the effectiveness of milking cluster disinfection procedures should be controlled regularly. Insufficient milking hygiene measures were also reported in another study analyzing MRSA transmission on Italian dairy farms [25].

Young stock and, in particular, calves, were shown to be a reservoir for LA-MRSA ST398 on dairy farms [11,13,19]. Waste milk, i.e., milk that for a range of reasons is not considered fit for human consumption, is frequently fed to calves on dairy farms. Thus, MRSA may be transferred to calves by feeding contaminated milk [26,27]. The detection of closely related LA-MRSA ST398 isolates in QMS and nasal swabs from calves on all dairy farms in our study clearly indicates a linkage of waste milk feeding to the appearance of LA-MRSA ST398 isolates in the nasal cavities of the calves. Even more impressive, the detection of closely related LA-MRSA ST398 isolates in QMS, nasal cavities of calves and suckers of automatic calf feeders on farm E illustrates the transmission of LA-MRSA ST398 during the feeding process.

In addition to LA-MRSA ST398 isolates from dairy samples, isolates from pigs were analyzed on farm F. Pigs are frequent nasal carriers of MRSA and a transmission from pigs to other animals was reported [17,19]. However, in our study, the LA-MRSA ST398 from pigs and the dairy barn showed clear genomic distinctions. A transmission of isolates only took place in close proximity between pigs and a heifer, which was kept in the pig barn.

Besides animal health, LA-MRSA ST398 may also pose a zoonotic risk for farm personnel [6]. However, the risk for severe human infections is considered to be low due to the loss of virulence-associated genes along with adaptation of *S. aureus* CC398 to livestock [8]. In our study, a transmission of LA-MRSA ST398 from animals to humans or vice versa was indicated on farms A, C and D. In particular, on farm C, closely related LA-MRSA ST398 isolates were detected in nasal swabs from a calf and the farm personnel as well as in BTM and QMS. This clearly underlines the zoonotic potential of LA-MRSA ST398 strains on livestock farms. Interestingly, on dairy farm A, a LA-MRSA ST398 isolate detected in one of three human nasal swabs was genetically distinct from all other isolates. Most likely, the affected person had acquired the strain on another occasion. Since farm personnel may change between livestock farms, it can be speculated that the detected LA-MRSA ST398 isolate was transmitted to the worker on another farm. Alternatively, further MRSA strains could be present on the farm but were not isolated, as we only sampled 30 of the 970 cows. The BTM isolate on this farm was also distinct from the majority of isolates, which also indicates that there may be more different types of LA-MRSA in this large herd.

Consumption of raw milk and raw milk products poses a risk for humans due to bacteria in the milk, originating either from the mammary gland of cows or from environmental contamination of the milk during the milking process. Food poisoning by enterotoxin producing *S*. *aureus* strains is one of these risks [28], others include infections with *Campylobacter* spp., occasionally leading to foodborne outbreaks or Shiga-toxin forming *E. coli* [29]. In our study, not all LA-MRSA ST398 isolates from BTM samples could be associated with isolates originating from QMS. In particular, on farm A, the LA-MRSA ST398 isolate from the BTM was distantly related to the isolates from QMS. This finding illustrates that standard BTM analysis on dairy farms may be reliable for detecting the presence of MRSA on dairy farms. However, more intensive sampling needs to be performed on the dairy farms to get a comprehensive overview about the MRSA presence and potential zoonotic risks. Looking at only one isolate from BTM, some potentially harmful MRSA strains may be underrepresented and remain undetected in the BTM.

This study shows that phylogenetic analyses of LA-MRSA ST398 transmission on livestock farms using WGS and cgMLST is suitable for tracking clonal strains among different sample types such as animals, humans and the environment on the farms. In contrast to studies in which *spa* or SCC*mec* typing was used for transmission statements, core genome analyses seem to be much more discriminative and give more coherent indications for the phylogenetic relationships, since LA-MRSA ST398 isolates may be genetically different despite being of the same *spa* and SCC*mec* type. This is also shown in the present study, in which some LA-MRSA isolates widely differed in their core genomes, although carrying the same *spa* type. However, the limitation of using cgMLST for tracking is the definition of a cluster of close relationship. It is uncertain, whether a cluster definition of ≤24 alleles difference gives the real image of the transmission events on the livestock farms, since the genome’s mutation rate in *S*. *aureus* is rather high compared to other bacteria [30]. Moreover, global MRSA distribution studies should be performed to analyze genomic distinctions among different strains from various sources with same *spa* or SCC*mec* types around the world.

## 5. Conclusions

Phylogenetic analyses using WGS and cgMLST are suitable for tracking the transmission of LA-MRSA ST398 across different niches on dairy farms. Feeding MRSA-contaminated waste milk to calves may be one cause for MRSA transmission to young stock. As a potential solution, waste milk might be heat-treated before feeding to reduce the MRSA burden. Moreover, insufficient hygienic measures during the milking time might lead to a transmission of MRSA between cows and poses a potential zoonotic risk. Improvement and regular control of hygienic procedures is highly recommended for preventing a spread of MRSA across livestock farms.

## Figures and Tables

**Table 1:** Overview of dairy herd sizes, number of retrieved LA-MRSA ST 398 isolates, source of isolation, *spa* and sequence type.

Farm	Dairy Herd Size	Number of LA-MRSA ^1^ Isolates	Source of LA-MRSA	*Spa* Types
A	350	9	BTM ^2^, QMS ^3^, TC ^4^, Calf, Human	t011, t2011
B	700	17	QMS, Calf, Heifer, Human	t034, t588, t19084
C	970	23	BTM, QMS, Calf, Human	t011, t034, t571
D	280	19	BTM, QMS, TC, Calf, Heifer, Human	t011, t034, t1928
E	94	25	BTM, QMS, TC, Calf, AF ^5^, Heifer, EN ^6^	t034
F	27	22	BTM, QMS, TC, Calf, Pig, EN	t011, t1451

^1^ LA-MRSA = livestock associated methicillin resistant *Staphylococcus aureus*; ^2^ BTM = bulk tank milk, ^3^ QMS = quarter milk sample, ^4^ TC = teat cup, ^5^ AF = automatic calf feeder, ^6^ EN = environmental sample.

## Data Availability

The assembled sequences of all MRSA isolates in our study are deposited in NCBI under the BioProject ID 634452.
